# LICEDB: light industrial core enzyme database for industrial applications and AI enzyme design

**DOI:** 10.1093/database/baaf001

**Published:** 2025-02-18

**Authors:** Lei Gong, Fufeng Liu, Chuanxi Zhang, Yongfan Ming, Yulan Mou, ZhaoTing Yuan, Haiming Jiang, Bei Gao, Fuping Lu, Lujia Zhang

**Affiliations:** School of Chemistry and Molecular Engineering, East China Normal University, No.500 Dongchuan Road, Minhang District, Shanghai, China, Shanghai 200062, China; School of Biotechnology, Tianjin University of Science and Technology, No.9, 13th Street, Economic and Technological Development Zone, Binhai New Area, Tianjin 300457, China; School of Biotechnology, Tianjin University of Science and Technology, No.9, 13th Street, Economic and Technological Development Zone, Binhai New Area, Tianjin 300457, China; Department of Micro/Nano Electronics, School of Electronic Information and Electrical Engineering, Shanghai Jiao Tong University, No.800 Dongchuan Road, Minhang District, Shanghai 200240, China; School of Automation, Central South University, No.932 Lushan South Road, Changsha City, Hunan Province, Changsha 418405, China; School of Chemistry and Molecular Engineering, East China Normal University, No.500 Dongchuan Road, Minhang District, Shanghai, China, Shanghai 200062, China; School of Chemistry and Molecular Engineering, East China Normal University, No.500 Dongchuan Road, Minhang District, Shanghai, China, Shanghai 200062, China; School of Life Science and Technology, Inner Mongolia University of Science and Technology, No.7 Alding Street, Kundulun District, Baotou City, Inner Mongolia, Baotou 014010, China; School of Biotechnology, East China University of Science and Technology, No.130 Meilong Road, Xuhui District, Shanghai 200237, China; School of Biotechnology, Tianjin University of Science and Technology, No.9, 13th Street, Economic and Technological Development Zone, Binhai New Area, Tianjin 300457, China; School of Chemistry and Molecular Engineering, East China Normal University, No.500 Dongchuan Road, Minhang District, Shanghai, China, Shanghai 200062, China; NYU-ECNU Center for Computational Chemistry, NYU Shanghai, No.268 Songlin Road, Pudong New Area, Shanghai 200062, China

## Abstract

Enzymes, serving as eco-friendly catalysts, are progressively supplanting traditional chemical catalysts in light industry sectors such as feed, papermaking, textiles, detergents, leather, and sugar production. Despite this advancement, the variability in the performance of natural enzymes and the fragmentation and diversity of existing data formats pose significant challenges to researchers. Furthermore, AI-driven enzyme design is limited by the quality and quantity of available data. To address these issues, we introduce the light industrial core enzyme database (LICEDB), the first database dedicated exclusively to managing and standardizing enzymes for light industry applications. LICEDB, with its integrated modules for data retrieval, similarity analysis, and structural analysis, will enhance the efficient industrial application of enzymes and strengthen AI-driven predictive research, thereby advancing data sharing and utilization in the field of enzyme innovation.

**Database URL**: http://lujialab.org.cn/on-line-databases/

## Graphical abstract

We introduce a database platform called LICEDB, which is dedicated to core enzymes in the light industry. It primarily collects three types of data from public databases, journal literature, and experimental data from research groups: basic information, experimental verification information, and industrial production information. LICEDB provides comprehensive performance parameters of light industry enzymes, offering a wealth of empirical data for enzyme design and production.

**Figure:**
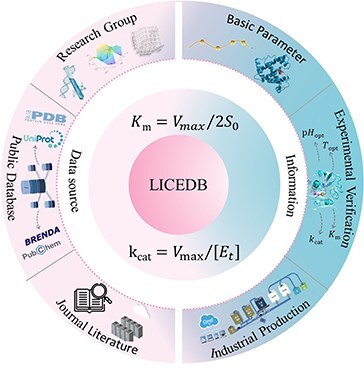

**Highlights**

- LICEDB is a database platform that provides core parameter information for the application and development of enzymes in the light industry.
- LICEDB contains three types of data information in the fields of feed, detergent, papermaking, textiles, leather, and sugar manufacturing: basic enzyme information, performance parameter information, and industrial application production parameter information, providing an empirical basis for industrial application and enzyme design, and improving efficiency.
- LICEDB provides functional modules such as data retrieval, upload, similarity analysis, and 3D structure visualization.

## Introduction

Light industrial enzymes refer to highly efficient biocatalysts utilized in light industry, renowned for their high catalytic activity, selectivity, and environmental adaptability [1]. These properties make enzymes ideal substitutes for traditional chemical and physical methods, particularly in green industrial processes such as starch hydrolysis, washing, and textile production [2]. Currently, enzymes and microbial cells are involved in ∼150 industrial processes, producing over 500 industrial products [3, 4], underscoring their pivotal role in advancing green industrial practices. However, natural enzymes often fail to meet the specific performance demands of certain light industrial processes, such as high activity, efficiency, and stability. Consequently, the modification of natural enzymes to develop high-performance variants suitable for specific environments has become a critical strategy in advancing the light industry.

Enzyme modification methods primarily include experimental screening techniques based on directed evolution and semi-rational design, as well as computational approaches grounded in physicochemical mechanisms. Zhu *et al*. [5, 6] employed directed evolution to enhance enzyme solvent stability in nonaqueous phases, developing industrially relevant enzyme variants such as lipase and polysaccharide monooxygenase. Tan *et al*. [7] improved the thermal stability of phytic acid enzymes from *Acidobacter* spp., tripling its half-life at 60°C and enhancing phosphorus utilization in feed production. Weidong Liu *et al*. [8] colleagues analyzed the structure of the Polyethylene terephthalate (PET)-degrading enzyme/sPETase complex through computational methods, yielding mutants with improved hydrolytic performance for PET. Munmun *et al*. [9] demonstrated the sustainable extraction of collagenase from leather waste via pineapple bromelain, achieving an 82.20% recovery rate. Despite the advances made by experimental and computational approaches, the vast potential of enzyme mutations and the inefficiency of the modification process present challenges. However, the data from these experimental and computational trials provide valuable insights for the industrial application and modification of light industrial enzymes, as well as abundant resources for data-driven intelligent enzyme design. Currently, AI-based design of enzymes relies on rich protein substrate information, such as protein sequences [10–13], protein structures [14–16], Enzyme Commission (EC） classification numbers [17, 18], *K*_m_ [19, 20], *k*_cat_ [21, 22], optimal temperatures [13, 23], substrate molecular maps [24, 25], and catalytic reaction equations [26, 27]. Park *et al*. [28] enhanced the catalytic efficiency and specificity of catechol-*O*-methyltransferase by designing functional domains that recognize specific substrates. Kroll *et al*. [21] curated and standardized 4271 enzyme sequences and molecular fingerprint data from BRENDA, SABIO-RK, and UniProt databases, training an accurate TurNuP model to predict enzyme activity parameters. However, existing literature largely focuses on positive results, with much valuable process experience data buried across various research teams, hindering full data sharing. Additionally, inconsistencies in data quality and a lack of standardized formats further limit their potential applications. Therefore, the systematic collection and management of light industrial enzyme data is essential for promoting industrial applications and AI-driven enzyme modifications.

Currently available modification data are often scattered across journal articles and laboratory records, with only a portion of it available in public databases. The BRENDA database, established in 1987 at the University of Braunschweig in Germany [29], houses extensive enzyme catalytic properties, substrate specificity, and reaction condition parameters, but the connection between experimental data and application fields remains weak, limiting its efficiency in supporting industrial applications. Other databases like BioFuelDB [30], UM-BBD [31], MetaBioME [32], dbMisLoc [33], ESOMIR [34], and HantavirusesDB [35] categorize enzymes by application field, but no specialized database exists for light industrial enzymes. Querying early research data on light industrial enzymes requires searching multiple databases or relevant literature individually, a method prone to omissions and inefficiencies, significantly hindering the development of light industrial enzyme research.

To address the limitations of existing databases, we have established the light industrial core enzyme database (LICEDB), which includes 48 235 core enzymes, providing comprehensive resources for the application, production, and modification of light industrial enzymes. LICEDB not only offers detailed information, such as protein sequences, structures, EC classification numbers, performance parameters, substrate molecular profiles, and catalytic reaction equations, but also serves as a data source for training machine learning models, promoting the development of enzyme performance prediction models, and enhancing the potential for data-driven enzyme modification and its application in light industries.

## Materials and methods

### Database architecture and web interface

LICEDB is a web-based database, and the entire service runs on a CentOS server (version 8.1, https://www.centos.org/). It utilizes MySQL (version 5.6, https://www.mysql.com/) as the data management system, featuring a stable and reliable structured design that efficiently meets various query requirements and enhances the data query speed and efficiency through index optimization. To ensure a user-friendly interactive experience, we designed the frontend using the Bootstrap framework (https://getbootstrap.com/) combined with HTML5, CSS3, and JavaScript technologies and incorporated jQuery (https://jquery.com/) as a utility library to construct a responsive web interface. This enables users to access databases on different terminal devices while ensuring visual consistency and interactivity across the interface. Additionally, we implemented the backend program using Python (version 3.6, https://www.python.org/) and Flask (version 1.1.2, https://flask.palletsprojects.com/), thereby providing efficient data processing and page-rendering mechanisms and facilitating rapid and flexible interactions with the database. Furthermore, LICEDB integrates AlphaFold2 (https://alphafold.com/) to predict protein structures [36], provides high-quality structural information for proteins in the database, and utilizes MolStar for the 3D visualization of protein structures [37]. Additionally, LICEDB incorporates external applications, such as BLAST (version 2.15.0) and Clustal Omega (version 1.2.4) [38], for analysis.

### Data collection and management

The goal of LICEDB is to compile enzyme information relevant to the light industry, primarily covering sectors such as starch hydrolysis, feed production, detergents, leather tanning, papermaking, and textiles. A detailed list of commonly used enzymes in each sector can be found in Table 1. We constructed a standardized molecular database of light industry enzymes by analyzing big data and the existing information on light industry enzymes. The types of detailed information we collected on light industry enzymes were as follows:

**Table 1.** The main fields of the light industry and the enzymes commonly used in production

Main fields of light industry	Industrial enzymes
Starch hydrolysis	α-Amylase, fungal α-amylase, β-amylase, glucoamylase, isoamylase, and pullulanase
Textile industry	Cellulases, amylases, catalases, pectinases, proteases, lipases, and laccases
Papermaking industry	Cellulase, hemicellulase, ligninase, amylase, lipase, pectinase, and laccase
Detergent industry	Protease, lipase, amylase, cellulase, mannanase, and pectinase
Leather industry	Lipase, protease, trypsin, papain, pepsin, acid protease, alkaline protease, and neutral protease
Feed production	Cellulase, phytase, protease, amylase, lipase, xylanase, pectinase, and hemicellulase

- enzyme names (name code, enzyme species in Chinese, enzyme species in English, and EC classification numbers);
- enzyme sources (species and family affiliation);
- sequence information (gene sequence, amino acid sequence, GenBank, or UniProt);
- structural information (RCSB Protein Data Bank (PDB) structure file, PDB number, and PDB information source);
- reaction environment information (engineered bacterial name, catalytic reaction equation, and enzyme activity measurement method);
- condition parameters (enzyme activity, optimum pH, optimum substrate, optimum temperature, *k*cat, and *K*m); and
- application sectors (starch sugar production, laundry, leather, papermaking, and textiles).

During the data collection phase, data sources were divided into three categories: the first category comprised existing experimental data produced in laboratories; the second category comprised data from public databases, including relevant data from the BRENDA [29], NCBI [39], EC-PDB, and KEGG ENZYME [40] databases; and the third category comprised data from recent literature published on light industry enzymes, which we obtained by searching the Scopus database using enzyme types combined with application fields as the search terms to identify relevant literature with these words in their title, abstract, and keywords. For example, the terms (TITLE-ABS-KEY((“*protease*” OR “*lipase*”) AND (“leather” OR “laundry”))) and (TITLE-ABS-KEY(“*amylase*” OR “glycosidase” OR “pullulanase”) AND (“sugar”)) were used. During the initial screening, we successfully gathered 1552 laboratory data entries, 10 023 public database entries, and 62 324 literature references. However, the inclusion criteria limited the selected papers to final published articles in journals, excluding review articles, book chapters, conference papers, books, notes, letters, and errata. The final number of included documents was 36 660.

To build a high-quality and standardized database, we will implement a rigorous data quality control strategy that includes data source management, data cleaning, standardization, and the use of automated tools. During data collection, we ensure that the data come from reliable sources and are collected using strictly designed methods. Specifically, we use Python-based automated scraping scripts to extract enzyme IDs, sequences, performance parameters, and other predetermined information from the data interface, storing the extracted data in local files for subsequent analysis and processing. In the data organization phase, we perform visual data analysis and cleaning using OpenRefine (version 3.5.2) [41]. The data cleaning process is as follows: (i) standardizing field names in the data tables to ensure uniform table structures; (ii) standardizing the data to ensure consistent formats and definitions: converting OpenRefine’s default settings to the text format of the imported file and converting text data into numerical data for analysis, while standardizing PDB IDs and sequence data to uppercase; (iii) data exploration: using “text facet” to explore the data dimensions of text columns, “numeric facet” to analyze data distributions, “timeline facet” to observe data over different time points, and “scatterplot facet” to analyze interactions between numerical variables, providing deeper insights into the data; (iv) removing duplicate data: using “custom facet” for custom filtering and filtering data by the “name code” field, deleting duplicates while retaining a single record and removing rows with blank “name code” values; (v) handling conflicting data: manually reviewing the reasonableness of conflicting data and using the mode strategy for data within reasonable ranges; (vi) filling missing data: employing semi-automated text mining algorithms from established databases such as RCSB PDB [42], EC-PDB, and UniProt [43] to fill in missing structural and sequence information. We also use a pretrained deep learning model DLKcat [22] suitable for LICEDB to supplement missing *k*_cat_ data. Before applying this model, we predicted *k*_cat_ data entries and assessed the model’s suitability for LICEDB by evaluating prediction errors to ensure the accuracy and reliability of the predicted data; (vii) data integration: merging all data tables from three sources. To enhance data processing efficiency and quality, we employ a modular decomposition approach to create reusable cleaning process packages, which are then automated via scripts, significantly reducing manual intervention and accelerating data processing. The final high-quality dataset, containing 48 235 entries, is stored in LICEDB, providing a reliable data source for future data science research.

## Results: database functionality

### Web interface

**Figure 1.**
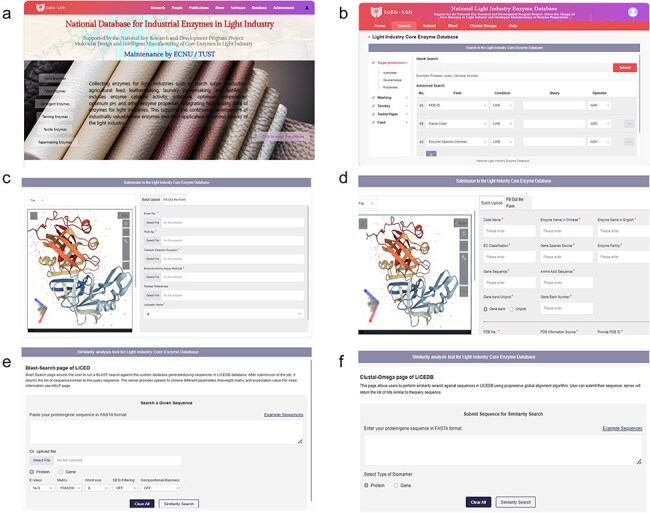
An overview of the LICEDB interface. (a) The homepage of the database, (b) the search interface of the database, (c) the batch data upload interface of the database, (d) the single data upload form interface, (e) BLAST similarity analysis tool, and (f) Clustal Omega similarity analysis tool.

To ensure researchers can understand how to use LICEDB quickly, the homepage of the website (Fig. 1a) provides an overview of LICEDB and quick entry to the initial registration and login page via the quick entry button (Supplementary Figure S1). After a successful login, users will be redirected to the retrieval interface (Fig. 1b), where they can begin to use LICEDB. In addition to the search function, LICEDB provides data submission (Fig. 1c and d), 3D visualization, similarity comparison analysis (Fig. 1e and f), and help functions. Users can quickly access other interfaces in the database by clicking on the navigation bar. In addition, we provided a side navigation bar (Fig. 1b) that records a list of commonly used enzymes in various industries involved in LICEDB, which helps users understand the classification overview information of LICEDB more intuitively.

#### Search function

**Figure 2.**
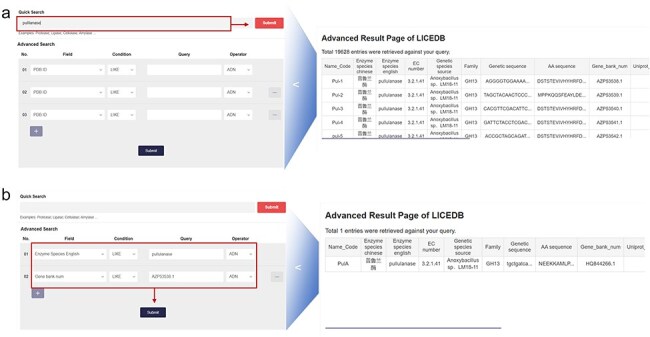
LICEDB search case. (a) Use the quick search feature to find information related to pullulanase and (b) use the advanced search feature to query for enzymes with the English name “pullulanase” and Gene Bank number “AZP53538.1” for related information.

In the search interface, we have provided two types of queries: (i) quick query: users can query all entries in the database that contain a certain keyword by typing it in; and (ii) advanced search: users can use all available database fields to filter the query results by setting up more complex criteria using Boolean expressions (e.g. AND, NOT, and OR), which allow users to precisely select the data they are interested in. We have provided a variety of filtering conditions in this function to allow users to add or delete filters to achieve specific conditions of record retrieval. In addition to the usual PDB ID keyword query, LICEDB also provides the name code, enzyme species in English, EC classification number, gene sequences, amino acid sequences, UniProt number, and GenBank number. For example, to query Pullulanase, we used the “Quick Search” module to search records related to the keyword “pullulanase,” clicked “Submit,” and retrieved 19 628 related records from the LICEDB. Using these records, we mainly searched for entries containing the keyword “pullulanase” in the field of Enzyme species in English (Fig. 2a) and used the “Advanced Search” module for the search. We set the filtering rules to “Enzyme Species English LIKE pulllanase” and “Gene bank num LIKE AZP53538.1” and clicked “Submit” to search and retrieve relevant data (Fig. 2b).

#### Analytical tool services

To enable users to understand the evolutionary relationship between Light industrial core enzyme sequences more efficiently and quickly, find similar sequences, and further analyze the structure, function, and evolution of target proteins, we added the sequence similarity search tool BLAST (Fig. 1e) and the multiple sequence comparison module Clustal Omega (Fig. 1f) to the LICEDB. Users can perform a similarity search based on all the sequences in the database by uploading a FASTA file containing a protein or nucleotide sequence. The program compares two or more protein or nucleic acid sequences using specific algorithms according to scoring rules to capture the optimal or maximum similarity match between sequences and generates a comparison result file. The outputs of the two tools contain information such as the similarity of the compared sequences, effective length of the comparison, number of mismatches, number of gaps, and comparison score. As an example, we performed a similarity analysis of hydrolase (PDB ID: 5URO) using the BLAST tool, and the output results are shown in Table 2.

**Table 2.** Comparison of 5URO proteins based on BLAST similarity analysis tool

Query sequence	Comparison sequence	Sequence similarity	Effective comparison length	Mismatch	*E*- value	Score
HYPJQ	Q7SHI0	38.329	347	197	7.21e-80	250
HYPJQ	P80299	31.624	351	192	2.24e-44	163
HYPJQ	Q6Q2C2	34.551	301	169	3.85e-43	159
HYPJQ	P34913	31.737	334	203	5.56e-43	159
HYPJQ	P34914	31.214	346	200	6.65e-42	156
HYPJQ	I6YGS0	29.167	312	196	6.98e-29	116
HYPJQ	O31581	26.384	307	176	5.37e-19	89.0
HYPJQ	Q6IE26	25.150	334	200	9.65e-19	89.4
HYPJQ	Q8IUS5	24.611	321	189	9.12e-18	86.7

As mentioned earlier, LICEDB serves as a useful resource for research on light industry enzymes because it constitutes a centralized and updated repository of information on enzymes used in light industries. Therefore, LICEDB welcomes users to submit new light industry enzyme data, and we have set up two data submission methods for this purpose, Form Submission (Fig. 1d) and Batch Submission (Fig. 1c), where users can submit their data records by clicking “Fill Out the Form” or “Batch Submission” in the “Submit” interface. For the “Fill Out the Form” or “Batch Upload” options, the required information items are marked with *, the information is reviewed to avoid errors; selecting the “Reset” button clears all the filled items and the “Submit” button completes the data upload after the information is completed.

It should be noted that when using the “Batch Upload” method, users should first click “Excel file model” to download the batch form, summarize the data, and then upload it to the LICEDB. Clicking “Batch Upload” submits the form and we will manually verify and check the data content after receiving the new submission. Once the validity of the data is confirmed, the database will be updated. If users have any questions about the LICEDB, they can click the “Help” button in the navigation bar to access the “Help” screen (Supplementary Figure S2), which provides an introduction to each module and a tutorial on how to use it. Additionally, users can contact us directly using the contact information provided on the page.

### Visualization of 3D structural windows

**Figure 3.**
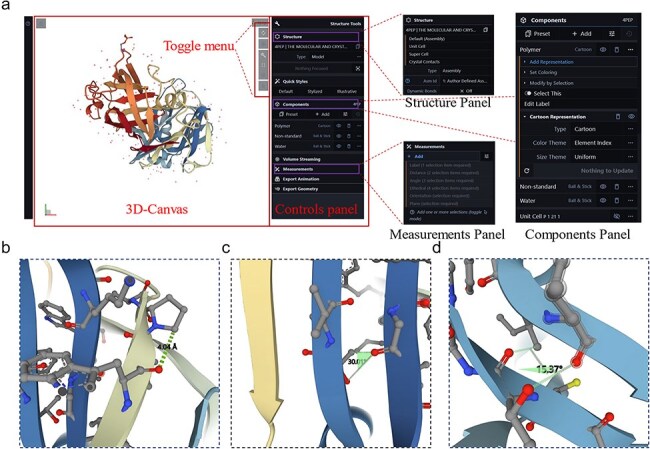
Interface details of LICEDB’s protein 3D structure visualization tool. (a) The 3D visualization interface of LICEDB consists of a 3D canvas, a switching menu and a control panel, as well as structure panels, component panels and measurement panels under the control panel details, (b) measuring distances to target atoms using the measurement panel, (c) measuring the angle of a target atom using the measurement panel, and (d) measurement of dihedral angles of target atoms using the measurement panel.

The 3D rendering of proteins is crucial for understanding their biological functions; therefore, a protein 3D viewer using the Molstar tool based on the advanced WebGL framework and Three.js 3D structure display library [20] was embedded in the sidebar panel of the server to allow visualization of enzyme structures. The interface consists of three modules: a 3D canvas, toggle menu, and control panel (Fig. 3a). Users can input the PDB ID into the protein viewer for systematic retrieval of the target PDB file or directly upload a PDB file by clicking “Open … .” The 3D structure of the protein appears on the 3D canvas and users can quickly access common operations of the 3D canvas through the toggle menu, such as taking screenshots, toggling the visibility of the right-side control panel, setting or controlling the 3D canvas information, and expanding the viewport. The rendering effects on the protein structure will be adjusted in the control panel, which includes the structure, components, and measurement panels (Fig. 3a). The structure panel offers various presentation forms of the structure, such as ball and stick, cartoon, Gaussian surfaces, labels, and molecular surfaces (Supplementary Figure S3). The measurement panel enables users to measure distances (Fig. 3b), angles (Fig. 3c), and dihedral angles (Fig. 3d) within the structure and displays the measurement results on the 3D canvas and in the corresponding control panel menu. The web-based 3D structure visualization technology embedded in the LICEDB does not require users to install additional software or plugins, allowing them to explore protein structures directly on the webpage.

### Overview of data collection

**Figure 4.**
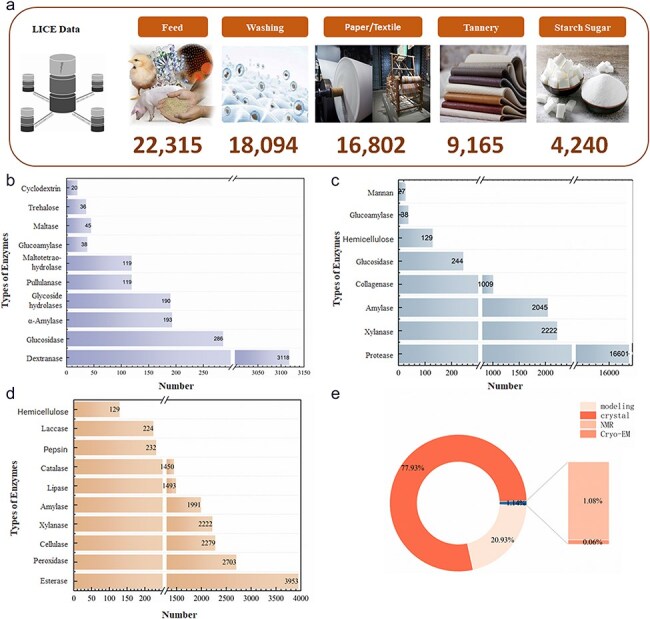
Statistical analysis charts of LICEDB. (a) Statistical composition of LICEDB data by application domains, (b) top 10 enzymes in the sugar-making domain in LICEDB, (c) top 10 enzymes in the feed domain in LICEDB, (d) top 10 enzymes in the papermaking domain in LICEDB, and (e) the source of enzyme structure information in LICEDB.

As of January 2024, 48 235 light industrial enzyme data entries were contained in the LICEDB, and mapping of the records to applications determined that enzymes for feed (46.26%) dominated the LICEDB, followed by enzymes for laundry detergents, paper/textiles, leather, and starch sugar production (Fig. 4a). Next, we grouped the records by EC values and calculated the number of entries in the LICEDB for seven categories of EC values (Table 3) and found that the data varied considerably, with hydrolytic enzymes and oxidoreductases dominating the LICEDB. Further analysis revealed that the most common hydrolytic enzymes in the LICEDB were proteases, amylases, and lipases, which are widely used in the feed sector (Fig. 4c), followed by cellulases, esterases, and xylanases, which are applied in the paper/textile industry (Fig. 4d), and dextranases, which are utilized in starch sugar production (Fig. 4b). Additionally, oxidoreductases, such as catalase and peroxidase, are widely used in the paper/textile field. Notably, 77.93% of the structural data of LICEDB were derived from X-ray crystallization, while the collected structural data derived from modeling accounted for 20.93% (Fig. 4e). This combination of experimental and computational data provides a richer and more comprehensive data resource, resulting in the LICEDB having higher data coverage and usability, which may aid future theoretical research and promote the application of light industrial enzymes.

**Table 3.** EC classification statistical data table for enzymes in LICEDB

EC number classification	Types of enzymes	Number of database entries
EC 1.X.X.X	Oxidoreductases	4036
EC 2.X.X.X	Transferases	2451
EC 3.X.X.X	Hydrolases	31 647
EC 4.X.X.X	Lyases	403
EC 5.X.X.X	Isomerases	48
EC 6.X.X.X	Ligases	26
EC 7.X.X.X	Translocases	51

### Case study

Here, we demonstrate the practical application of LICEDB through a case study on the thermal stability modification of pepsin. Pepsin is a classic model protein in aspartic protease research, widely used in the hydrolysis of large molecular protein substrates, particularly in feed processing and leather manufacturing industries. To explore potential high thermal stability variants of pepsin, we used the LICEDB platform to quickly retrieve pepsin sequences from various sources, analyze their homology, and perform mutation design based on this information, thereby accelerating the screening and optimization of high-performance enzymes.

First, we accessed the “Quick Search” feature by clicking the “Search” button at the top of the homepage, using “pepsin” as the keyword to search for relevant records in the database, and retrieved typical pepsin sequences from different sources (Supplementary Figure S4A). Next, using the BLAST tool provided by the LICEDB platform, we aligned the sequences and assessed the homology of the pepsin sequences, selecting the highest-scoring protein (PDB ID: 4PEP). We then used the visualization tools in the “Submit” interface to analyze the conserved domains of the 4PEP protein (Supplementary Figure S4B), identifying key sites that may influence its thermal stability. Based on this information, we designed mutations and experimentally validated their changes in half-life. After iterative optimization, we obtained the S129A mutant, which showed a 66.3% increase in half-life at 70°C.

## Conclusion

We have developed LICEDB, the first database dedicated to the performance parameters of light industrial enzymes. This database systematically collects fundamental information, experimental validation, and industrial application data of light industrial enzymes, providing a comprehensive resource to support their industrial use. LICEDB serves not only as a repository of data but also as a catalyst for innovation, enhancing the predictive accuracy of AI models for enzyme modifications and thereby facilitating their efficient application in industrial processes. To ensure the database remains current and accurate, we have integrated Change Data Capture technology with a scheduled incremental synchronization strategy for daily updates. This system captures and verifies data in real time against predefined constraints in our data tables, ensuring data integrity and consistency. Once validated, the Database Management System automatically executes updates through its task scheduling, ensuring that LICEDB remains a reliable and cutting-edge resource in the field of industrial enzyme research, driving the advancement of enzyme design and application.

## Supplementary Material

baaf001_Supp

## Data Availability

LICEDB can be accessed online at http://lujialab.org.cn/on-line-databases/, and is currently under embargo for 12 months from the date of publication of the article, or can be provided upon reasonable request.
