# Supplementary material for: LICEDB: light industrial core enzyme database for industrial applications and AI enzyme design
Source: Database (Oxford). 2025 Feb 18;2025:baaf001. doi: 10.1093/database/baaf001 (PMC11842304; doi:10.1093/database/baaf001)
Supplement: baaf001_Supp [file baaf001_supp.zip › suppl_data/03. Supplementary Material.docx]

**Supplementary Material**

**Fig. S1.** The initial registration and login interface of LICEDB.

**Fig. S2.** Details of the LICEDB help interface.

**Fig. S3.** LICEDB provides different 3D representations of protein structures.

**Fig. S4.** The LICEDB Case Study on the Thermal Stability Modification of Pepsin.

**
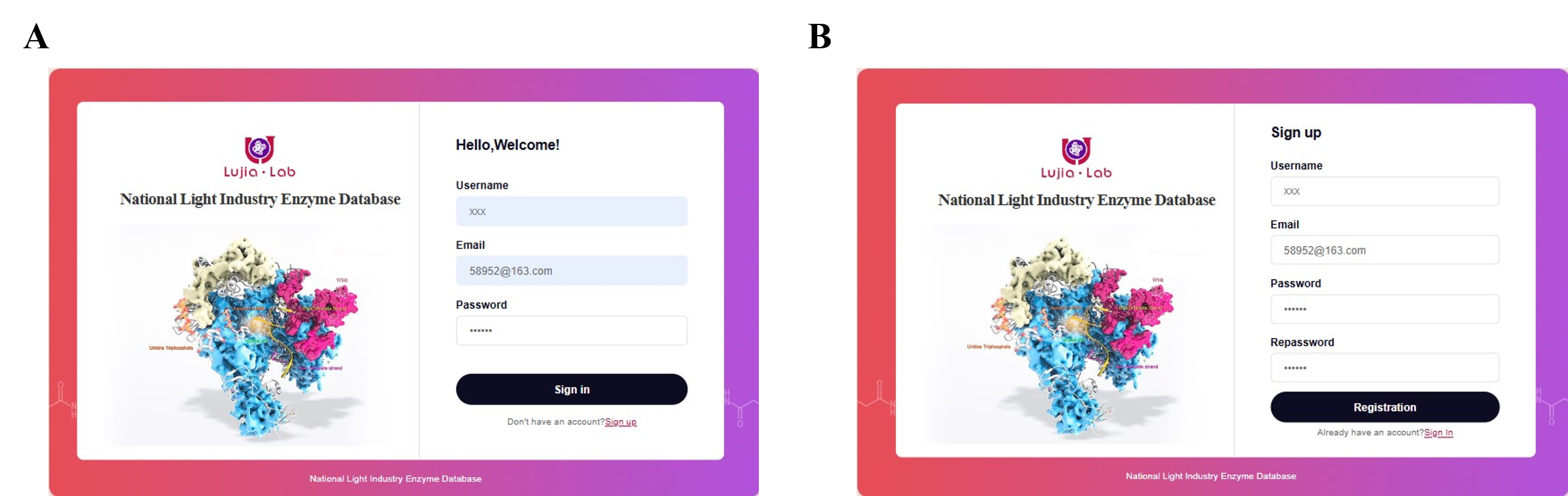
**

**Fig. S1.** The initial registration and login interface of LICEDB. (A) LICEDB login interface. (B) LICEDB register interface.


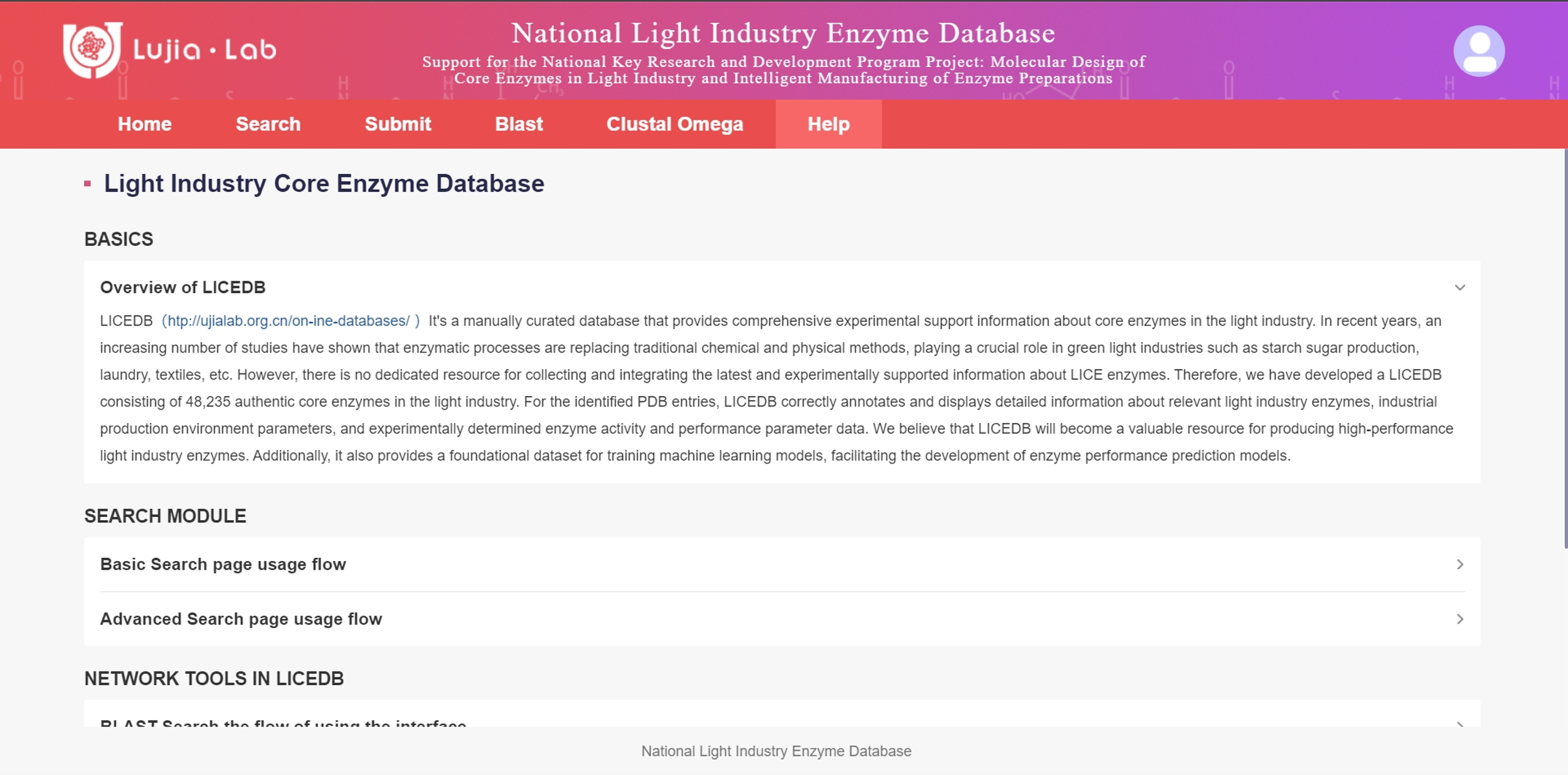


**Fig. S2.** Detailed information about the LICEDB help interface. The help interface mainly covers assistance for the search module, online tools module, and data upload module, with detailed information available by clicking on the respective modules.


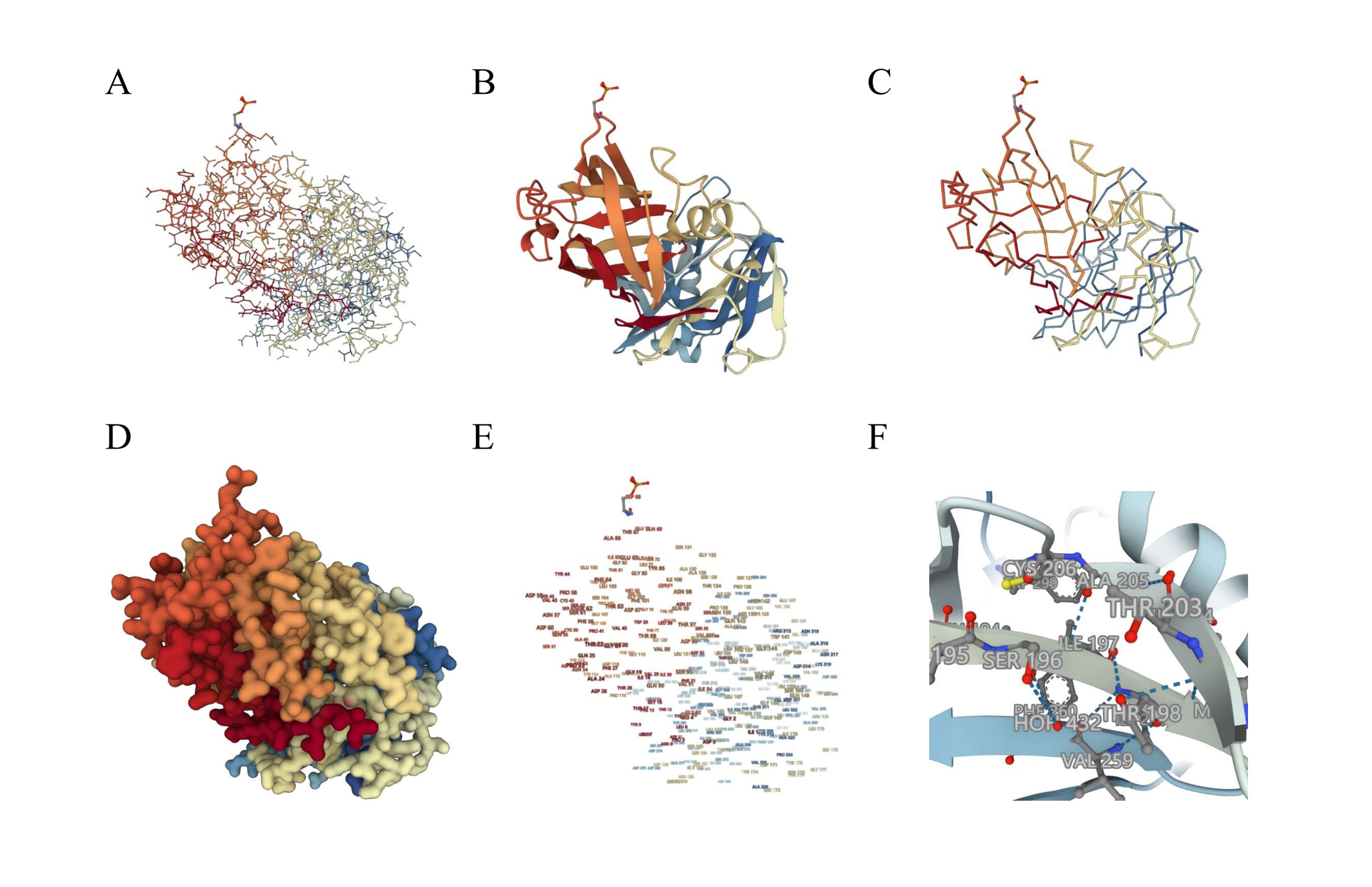


**Fig. S3.** Different 3D Visualization Rendering Formats for Protein 4PEP on the LICEDB Platform. (A) 4PEP structure Ball & stick diagram. (B) 4PEP structure Cartoon diagram. (C) 4PEP structure Backbone diagram. (D) 4PEP structure Gaussian surface diagram. (E) 4PEP structure Label diagram. (F) 4PEP structure Combined display of Cartoon and Label diagram.


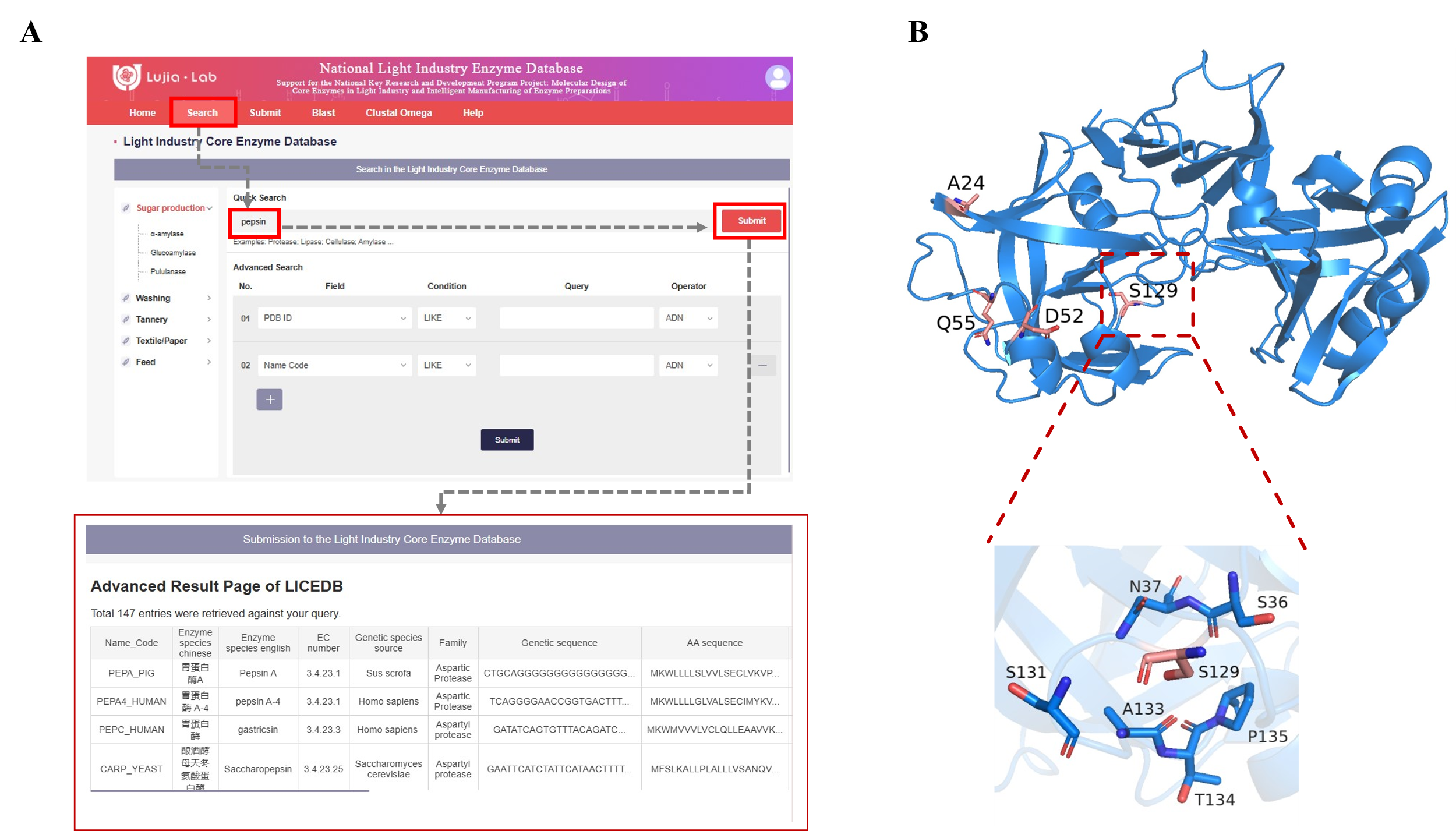


**Fig. S4.** The LICEDB Case Study on the Thermal Stability Modification of Pepsin. (A) The Process and Results Display of Pepsin Retrieval in LICEDB. (B) 3D Visualization of the Most Conserved 4PEP Protein in LICEDB, with the Mutated Structural Domain of the Key Mutation Point A133.
